# Reduction of Flicker in Four-Stroke Motion of Color Images

**DOI:** 10.1177/2041669517750400

**Published:** 2018-01-11

**Authors:** Takahiro Kawabe, Shin’ya Nishida

**Affiliations:** NTT Communication Science Laboratories, Kanagawa, Japan

**Keywords:** higher order motion, local motion, motion

## Abstract

When two sequential video frames extracted from a single video clip are followed by the negative of the two frames, a viewer often experiences a visual illusion whereby a scene in the frames continuously moves in a single direction (four-stroke apparent motion). To create a four-stroke apparent motion display, the image intensities of the whole of the second pair of images are reversed. However, this intensity reversal creates a strong impression of flicker that can be undesirable for comfortable viewing. This study reports that four-stroke apparent motion can be induced by only reversing the luminance intensities in those spatial areas which contain motion signals in high-pass filtered images. This use of only a partial reversal of image intensities greatly reduces the apparent flicker in the display while retaining motion perception.

When a sequence of two video frames in which, say, a bright bar moves to the right are presented repeatedly, a viewer perceives an oscillation of the bar (Figure 1(a)). However, when the two video frames are followed by the same frames with the luminance polarity reversed (Figure 1(b)), the viewer perceives the motion of the bar as being in a consistent direction. This sort of illusory apparent motion is called four-stroke apparent motion (Anstis & Rogers, 1986; Mather, 1991). Four-stroke apparent motion displays stimulate the detector of the spatiotemporal image flow as shown in Figure 1(c). Because the movement of bright and dark bars, respectively, falls on the positive and negative lobes of the detector’s receptive field, a viewer perceives a consistent rightward motion.

**Figure 1.**
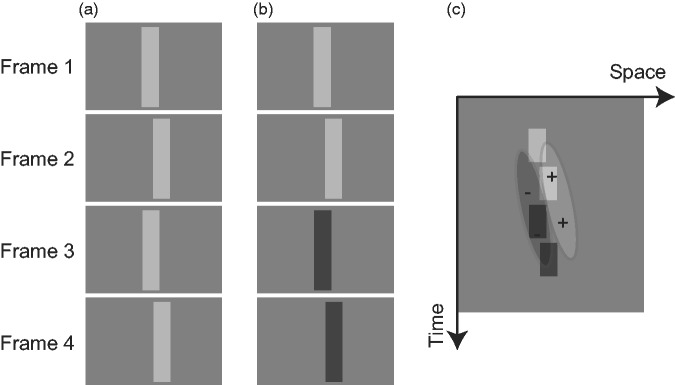
(a) A sequence of video frames causing an apparent back-and-forth motion. (b) A sequence of video frames causing apparent unidirectional motion. (c) A schematic diagram to explain how a four-stroke apparent motion display stimulates the detectors of the spatiotemporal energy flow.

Those who want to add four-stroke motion to a natural image will normally try to reverse the intensities of complete image areas of all intensity channels. However, such an intensity reversal results in an intensive flicker, which is not always desirable for comfortable viewing.

We aimed at reducing the apparent flicker by omitting those intensity reversals which were redundant. We focused on three redundant components: motion components at low spatial frequency, chromatic components, and static components. In four-stroke motion, unlike the object’s edges, the uniform surface of a slowly moving object does not always contain effective image motion signals across two video frames. Thus, motion at low spatial frequencies, which are related to image information at the surface, may not contribute to four-stroke apparent motion, and so may be omitted. In addition, it is well known that chromatic components do not strongly contribute to motion perception (Kawabe, Fukiage, Sawayama, & Nishida, 2016; Ramachandran & Gregory, 1978), and hence the reversal of chromatic components may not be necessary for producing four-stroke apparent motion of color images. Finally, in the previous method, intensity reversals are applied even to static regions, which may not contribute to motion perception. Thus, we should apply intensity reversals only to those spatial regions that contain motion signals.

Figure 2 shows the processing pipeline of our technique. Our technique aims at converting an original frame (Figure 2(a)) into an image with luminance intensity reversals in spatial areas with motion signals in a high-pass filtered image (Figure 2(b)). First, we obtain color and luminance channels of the original image by converting the RGB color space of the frame into, for example, CIE LAB (Figure 2(b)). The color channel image (Figure 2(c)) is kept intact. The image in the luminance channel is further decomposed into low-pass (Figure 2(d)) and high-pass (Figure 2(e) and (f)) filtered images. The cut-off frequency is a free parameter that users need to determine based on the magnitude of displacement in the sequence: Larger displacements require lower cut-off frequencies. Next, we determine motion and static areas on the basis of an optical flow calculation between the two frames (Figure 2(g) and (h)). Areas having a motion velocity that is higher or lower than 1 (pixels) are considered as static and motion areas, respectively. In Figure 2(e) and (f), static and motion areas are given values of 0 and 1, respectively. To apply intensity reversals only to motion areas, we multiply the intensity-reversed high-pass image (Figure 2(e)) by the motion areas and the unchanged intensity high-pass image (Figure 2(f)) by the static areas. By combining the color channel image (Figure 2(c)), the low-pass filtered image (Figure 2(d)), and the manipulated high-pass filtered images (Figure 2(e) and (f)), an image with luminance intensity reversal in the motion areas of the high-pass filtered image (Figure 2(b)) is obtained. The resulting image is more similar to the original image (Figure 2(a)) in comparison with an image that has undergone intensity reversals over the total image area and for all intensity channels (Figure 2(i)).

**Figure 2.**
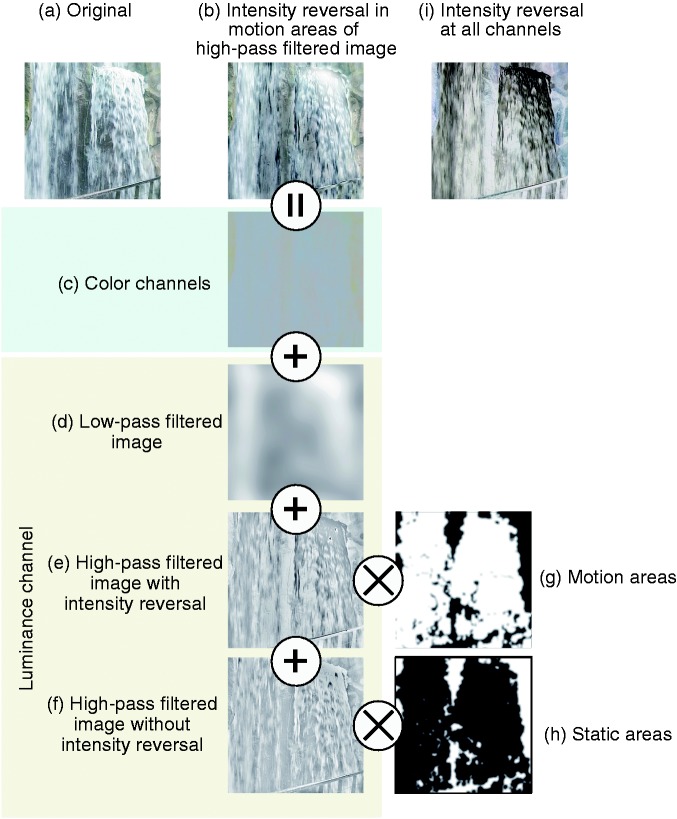
A schematic explanation of our method of creating an intensity-reversed video frame for four-stroke apparent motion. (a) Original, (b) intensity reversal in motion areas of high-pass filtered image, (c) color channels (d) low-pass filtered image, (e) high-pass filtered image with intensity reversal, (f) high-pass filtered image without intensity reversal, (g) motion areas, (h) static areas, and (i) intensity reversal at all channels.

Examples of the resultant four-stroke motion displays are shown in Videos 1 and 2, each of which shows a comparison with the original four-stroke motion displays. In an experiment, we presented, side by side, one of clips and the clip played backward on a CRT display and asked each of nine observers to answer the following questions by pressing assigned keys. For Video 1, the question was “which of waterfalls went downwards?” and for Video 2, the question was “which of the two soap bubbles went upwards?” Each of clips was tested 20 times. We tested four clips in total. The results showed that the proportion of observations identifying a correct clip was 0.948 on average with the standard deviation across the observers of 0.052. The same nine observers also compared the subjective magnitude of flicker between a video created using an original four-stroke motion and one created using our technique. All of the observers reported that the videos created with our technique had weaker flicker than those with the original four-stroke motion.

By combining the various modifications, our technique provides a four-stroke apparent motion with reduced flicker without hampering motion perception. Our technique could, possibly, be of use to vision science researchers who want to clarify how color and luminance are integrated in motion processing. However, the determination of the cut-off frequency for the high-pass filter is left as an open issue for future study. In addition, our technique does not work with a long observation distance because motion at a relatively high spatial frequency bands is critical here. Even with these limitations, our technique provides a strong tool for visually representing continuous motion in a more comfortable and attractive way.

## Supplementary Material

Supplementary material

Supplementary material
